# Targeting Gut Microbiota as a Novel Strategy for Prevention and Treatment of Hypertension, Atrial Fibrillation and Heart Failure: Current Knowledge and Future Perspectives

**DOI:** 10.3390/biomedicines10082019

**Published:** 2022-08-19

**Authors:** Oxana M. Drapkina, Adel A. Yafarova, Anastasia N. Kaburova, Anton R. Kiselev

**Affiliations:** National Medical Research Center for Therapy and Preventive Medicine, 101990 Moscow, Russia

**Keywords:** gut microbiota, hypertrophy, fibrosis, remodeling, hypertension, atrial fibrillation, heart failure, modulation

## Abstract

Cardiovascular diseases (CVDs) remain the major public health concern worldwide. Over the last two decades, a considerable amount of literature has been published on gut microbiota (GMB) composition and its metabolites, involved in the pathophysiology of CVDs, including arterial hypertension, atrial fibrillation, and congestive heart failure. Although many types of medicines are available to treat CVD, new therapeutic tools are needed to improve clinical outcomes. A challenge that often arises in the researchers’ community is how to manipulate the GMB to manage cardiovascular risk factors. Therapeutic strategies designed to manipulate GMB composition and/or its metabolites include dietary approaches, prebiotics/probiotics supplementation, and fecal microbiota transplantation (FMT). In this review, we have focused on three main cardiovascular pathologies (arterial hypertension, atrial fibrillation and heart failure) due to their shared common pathophysiological pathways and structural changes in myocardium, such as inflammation, hypertrophy, fibrosis, and myocardial remodeling. The main aims of the review are: (1) to summarize current knowledge on the key pathophysiologic links between GMB and CVDs, and (2) discuss the results of the studies on GMB modulation for the prevention and treatment of selected CVDs.

## 1. Introduction

Cardiovascular diseases (CVDs) remain the major public health concern worldwide. The Global Burden of Disease Study 2019 has shown that CVDs account for one-third of all deaths worldwide [1]. As the incidence of CVDs worldwide reaches alarming levels, there has been renewed interest in developing new preventive and therapeutic approaches. The gut microbiota (GMB) has long been a question of great interest in a wide range of health issues, in particular CVDs. The challenge faced by many researchers is that cardiovascular events could not be fully explained by the so-called conventional cardiovascular risk factors [2]. In this regard, gut microbiota dysbiosis, high exposure to endotoxin, and an increased trimethylamine N-oxide (TMAO) level represent novel possible risk factors for CVDs along with abnormal sleep duration, genetic markers, air pollution and environmental noise. The major advances in high-throughput sequencing technology and metabolomics have explored the pivotal role of GMB and its metabolites in regulating both inflammatory and fibrotic responses and further cardiac remodeling in a host organism [3].

The GMB metabolites, such as short-chain fatty acids (SCFA), TMAO, bile acids (BAs), certain uremic toxins, and lipopolysaccharide (LPS) exert multiple distinct effects on the occurrence and development of CVDs which are discussed further. Short-chain fatty acids (SCFA), particularly acetate, propionate and butyrate, are beneficial GMB-derived metabolites which are formed through bacterial fermentation of dietary fiber. Short-chain fatty acids also could be ingested in the diet or synthesized in the human body by certain metabolic processes such as fat oxidation during fasting-associated ketosis or after alcohol consumption [4]. Trimethylamine N-oxide is one of the harmful metabolites to the host organism which is formed from carnitine-, choline-, and betaine-rich foods, such as egg yolk, red meat, and certain seafoods, through the action of TMA-lyase in gut microbes, followed by oxidation by hepatic flavin monooxygenases [5]. Lipopolysaccharide is an endotoxin found in the outer layer of Gram-negative bacteria (primarily *Escherichia* genera) which has been established to boost inflammatory response in the host organism [6].

Recent evidence has shed light on the therapeutic potential of the modulation of both GMB and microbial metabolites in selected CVDs. The main therapeutic strategies designed to manipulate GMB composition and/or its metabolites include dietary approaches, prebiotics/probiotics supplementation, inhibition of TMAO formation, direct administration of SCFA, and fecal microbiota transplantation (FMT). Prebiotics are substances that induce the growth or activity of microorganisms contributing to the well-being of their host [7]. Probiotics are live microorganisms that, when administered in adequate amounts, exert a health benefit on the host [8]. Fecal microbiota transplantation (FMT) is an intervention to restore the GMB of patients by transfer of specifically prepared stool samples from healthy donors to recipients [9]. Manipulating both GMB composition and metabolism may become a key target for preventive and curative interventions for CVDs. Notably, we intentionally skipped antibiotic use as an option for GMB modulation due to the spread of antibiotic-resistant bacteria and the high risk of severe complications such as *C. difficile* infection.

Although coronary artery disease and type 2 diabetes mellitus have also been proven to affect cardiac remodeling, these topics are beyond the scope of our review. To date, there is a huge amount of data, concerning the role of GMB in the pathogenesis and progression of type 2 diabetes mellitus and coronary heart disease (3971 and 312 publications over the last 5 years, respectively) [10,11]. To our opinion, these topics are well described elsewhere [12,13]. In contrast, the number of publications on the role of the intestinal microbiota in the pathogenesis of chronic heart failure, atrial fibrillation and arterial hypertension is relatively limited.

In this review, we have focused on three main cardiovascular pathologies (arterial hypertension, atrial fibrillation and heart failure) due to their shared common pathophysiological pathways, such as inflammation, hypertrophy, fibrosis, and myocardial remodeling. The main aims of the review are: (1) to summarize current knowledge on the key pathophysiologic links between GMB and CVDs, and (2) discuss the results of the studies available to date on GMB modulation for the prevention and treatment of selected CVDs.

## 2. Arterial Hypertension

### 2.1. Gut Microbiota and Its Metabolites Contribute to Blood Pressure Regulation

#### 2.1.1. Possible Links between Gut Microbiota Composition and Arterial Hypertension

The accumulating evidence shows that GMB has been implicated in the regulation of blood pressure (BP) and the development of arterial hypertension (HTN). In clinical studies, pre-hypertensive, hypertensive individuals and their counterparts showed distinct differences in GMB composition with regard to several bacterial taxa [14]. Numerous large cross-sectional studies have attempted to describe the relationship between GMB and HTN development in humans [14,15,16,17]. The FINRISK 2002 study has established that 45 microbial genera and 19 *Lactobacillus* species were linked to BP indices. These results need to be interpreted with caution as the associations between overall GMB composition and BP were weak [15]. In the CARDIA study (Coronary Artery Risk Development in Young Adults), gut microbial diversity and the abundance of the *Robinsoniella* genus were negatively associated with systolic BP [16]. However, the HELIUS study (Healthy Life in an Urban Setting) suggested that GMB alterations may explain only 4.4% and 4.3% of the variability in systolic and diastolic BP, respectively [17]. These findings may be somewhat limited by 6 distinct ethnic groups and possible interference of certain comorbidities such as diabetes and chronic kidney disease, therefore the results could not be extrapolated to all patients.

Several lines of evidence suggest that GMB dysbiosis promotes angiotensin-II (Ang-II) induced HTN and HTN-related organ damage [18,19]. Karbach S. et al. (2016) established that germ-free (*GF*) mice were protected from Ang-II-induced *BP* increase and vascular dysfunction. In a study of mice injected with Ang-II, they have found that GF- mice had lower BP and less cardiac fibrosis in comparison to those who underwent FMT before the experiment or sham GF group [18]. FMT from spontaneously hypertensive rats (SHRs) to normotensive Wistar-Kyoto rats raised BP in the recipients [20]. 

#### 2.1.2. Gut Microbiota Contributes to Salt-Sensitivity of Blood Pressure

**Figure 1:**
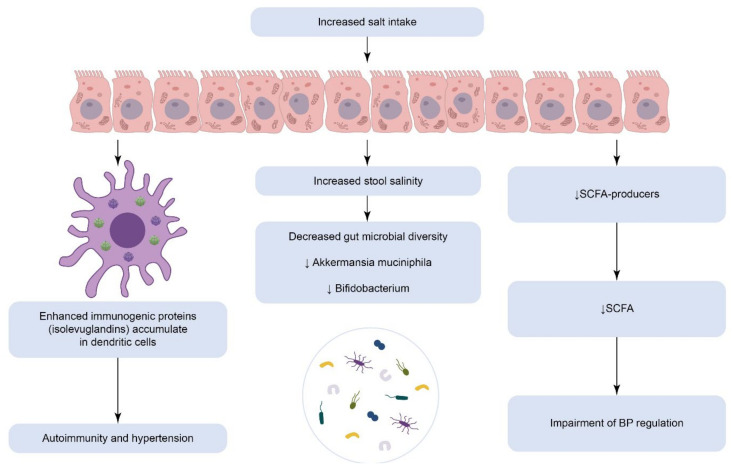
The impact of excess salt intake on gut microbiota composition and arterial hypertension development. BP: Blood Pressure, SCFA: Short Chain Fatty Acids.

A high-salt diet is pointed to as one of the strongest risk factors for CVDs due to the presence of salt sensitivity of blood pressure (SSBP) which has been linked to increased cardiovascular events and reduced survival [21]. The pathogenesis of the SSBP phenomenon is quite complex and involves GMB along with immune cells, kidneys, vasculature, and the central nervous system. As shown in Figure 1, GMB seems to be an integral moderator in the uptake of salt in the host organism and may respond to salt sensitivity in HTN.

The proximal colon serves as the main site of dietary sodium absorption and plays an important role in BP regulation [22]. In a study of a rat model, Linz B. et al. (2016) showed that inhibition of the gut sodium–hydrogen exchanger 3 led to BP reduction and increased urine sodium excretion [23]. In salt-sensitive animal models, it has also been conclusively shown that high sodium consumption decreases both microbial diversity and short-chain fatty acids (SCFAs) production [24]. Data from another study have identified a reduction in GMB diversity and depletion in *Akkermansia muciniphila* and *Bifidobacterium* associated with increased stool salinity [25].

In a study conducted by Ferguson J.F. et al. (2019), there has been described a novel GMB-related mechanism how an excess sodium intake contributes to inflammation and HTN. They have revealed that a high-salt diet causes an increased abundance of pro-inflammatory intestinal microbes and is associated with enhanced immunogenic proteins adduct formation in antigen-presenting cells. These highly reactive proteins such as isolevuglandins accumulate in dendritic cells and act as neoantigens, driving autoimmunity and HTN [26]. This finding broadly supports the work of other studies in the area linking GMB composition, excess sodium intake and elevated BP.

### 2.2. Gut Microbiota Modulation Approaches in Arterial Hypertension Management: From Dietary Interventions to Washed Microbiota Transplantation

#### 2.2.1. Dietary Interventions

The maintenance of healthy «normotensive» GMB characterized by increased microbial diversity and richness of SCFAs—producers, lies at the core of the BP-lowering effect related to dietary interventions. Chen L. et al. (2020) conducted a randomized controlled trial (RCT) to explore whether a decrease in salt intake alters GMB composition and serum SCFA levels among untreated hypertensive subjects. Restricting sodium intake to 2000 mg per day for 6 weeks resulted in a statistically significant increase in SCFAs (including 2-methylbutyrate, butyrate, hexanoate, isobutyrate, and valerate), which are beneficial GMB-related metabolites known to induce vasorelaxation. The most important clinically relevant finding from this trial was that increase in plasma SCFA levels was associated with BP reduction and improvement of arterial compliance [27].

A systematic review and network meta-analysis of 67 trials enrolling 17,230 participants have demonstrated that dietary approaches to stop hypertension (DASH) such as the Mediterranean diet (MedD), low carbohydrate diet and some others had more pronounced BP-lowering effects as opposed to the usual diet [28].

In a study of rat models, Shi H. et al. (2021) investigated the effects of intermittent fasting (IF) on the GMB, bile acids (BAs) metabolism and BP regulation. At baseline, the concentrations of several BAs in SHRs were significantly lower than in Wistar Kyoto rats. These findings may indicate that BAs serve as important ligands for host nuclear receptors and/or G-protein coupled receptors, which have a pivotal role in BP regulation. In SHRs, it has also shown been that the reshaping of GMB led to increasing plasma BAs with subsequent reduction of systolic BP [29].

In a prospective observational study conducted in a cohort of 1422 subjects, it was shown BP-lowering effect of IF. The results indicated a significant BP decrease (from 131.6 ± 0.7 to 120.7 ± 0.4 for systolic BP and from 83.7 ± 0.4 to 77.9 ± 0.3 for diastolic BP; *p* < 0.001) whereby mean values did not drop below the normal range. The reduction in systolic and diastolic BPs was more significant in participants who fasted longer with no gender difference, stabilizing for the whole cohort at around 120 and 78 mmHg, respectively [30].

Maifeld A. et al. (2021) attempted to evaluate the impact of fasting on cardiometabolic effects by comparing a modified DASH-diet (fasting + DASH-diet) and DASH-diet only. According to results, both DASH-diet and fasting + DASH-diet reduced office systolic BP after 3 months post-intervention. It was also found that participants who followed fasting + DASH-diet achieved a sustained 24-h ambulatory systolic BP reduction, as opposed to those who followed DASH-diet alone. The unexpected finding was that that fasting + DASH-diet induced distinct GMB and immunome changes that were related to starvation itself, leading to a constant BP benefit, which was not observed in the comparison group. Notably, patients in the group fasting + DASH diet significantly reduced their intake of antihypertensive medication by 3 months post-intervention, compared to subjects following a DASH diet only [31]. Thus, obtained results suggest that IF may serve as an effective tool to improve anti-hypertensive treatment.

In different patient cohorts, it has been shown that MedD positively influences the GMB via enhancement of microbial diversity, a feature that has a health-promoting effect [32,33]. Garcia-Mantrana et al. (2018) pointed out that a higher adherence to the MedD is characterized by an increase in *Bifidobacteria*, lower *Firmicutes-Bacteroidetes* ratio and a higher percentage of SCFAs [34].

#### 2.2.2. Prebiotics and Probiotics Supplementation

Previous research findings regarding the BP-lowering effect through GMB modulation with prebiotics/probiotics have been inconsistent and contradictory [35,36]. It is well-known that fermentable dietary fibers constitute the most important substrate for the GMB to produce SCFA. In animal models, Marques F.Z. et al. (2017) found that high dietary fiber consumption induced an increase in the abundance of acetate-producers. The work has also revealed that a high-fiber diet and acetate supplementation were responsible for the decrease in the *Firmicutes/Bacteroidetes* ratio and a concomitant increase in the prevalence of *Bacteroides acidifaciens*. The study has also confirmed that reshaping of GMB led to a significant reduction in systolic and diastolic BPs and attenuation of both cardiac fibrosis and left ventricular hypertrophy [37].

In a meta-analysis of 23 RCTs, there has been reported a mild BP-lowering effect of several types of viscous fiber supplementation (β-glucan from oats and barley, guar gum, konjac, pectin and psyllium) in hypertensive patients [38]. However, these findings need to be interpreted with caution because systolic BP reduction was confirmed only for using psyllium fiber. Another meta-analysis that set out to evaluate the effect of prebiotic inulin-type carbohydrate (ITC) supplementation on both systolic and diastolic BPs, did not show any significant differences. According to the results, the addition of ITC did not have an overall lowering effect on systolic and diastolic BP, while subgroup analysis showed that ITC can decrease systolic BP only in women [39].

Pooled results from the meta-analysis of 23 RCTs have demonstrated that modulation of GMB through probiotics intake led to a reduction in both systolic and diastolic BP by 3.05 mmHg and 1.51 mmHg, respectively, as compared with controls [40]. Interestingly, the efficacy of probiotic supplementation was similar to such lifestyle interventions as a dietary salt reduction of <2 g per day and resistance training [41]. Moreover, the probiotic-mediated attenuation of systolic BP was observed only in patients with HTN or type 2 diabetes, while the decrease in diastolic BP was reported only in HTN. However, even a small reduction in BP may have important public health benefits and cardiovascular consequences. The BP-lowering effect reported by the current meta-analysis was modest and could last only for a short period of time (8–10 weeks) [40]. Furthermore, a clinical study conducted by Aoyagi, Y. et al. (2017) has demonstrated that frequent intake of *Lactobacillus casei* strain *Shirota* fermented milk products decreased the likelihood of HTN manifestation in elderly normotensive people over a 5-year period [42].

It is worth mentioning that a previous systematic review and meta-analysis had also suggested optimal threshold of duration (>8 weeks) and daily dose (probiotics >1011 colony-forming units), and recommended prescribing multiple bacteria for more effective intervention [43].

#### 2.2.3. Postbiotics (e.g., SCFAs)

Numerous studies have demonstrated that SCFA take part in BP regulation and supplementation with SCFA could reduce BP through anti-inflammatory and vasodilatory mechanisms [44,45]. In animal experiments, the administration of acetate has been shown to reduce BP. Acute infusion with propionate also provided an immediate BP-lowering effect in the mice model. The addition of propionate to drinking water lowered BP and attenuated cardiac damage in Ang-II-infused hypertensive mice [46]. Butyrate infusion has been demonstrated to shift the GMB composition and mitigated Ang-II induced HTN in mice thereby improving cardiac and vascular function [47]. Chronic SCFA supplementation also can reduce BP and improve cardiovascular outcomes [48]. Kaye D.M. et al. (2020) found that supplementation with all 3 main SCFAs could alleviate HTN and its complications in the Ang-II model even in the absence of dietary fiber [49]. To date, the direct effect of SCFAs administration on BP levels in humans has not yet been evaluated.

#### 2.2.4. Washed Microbiota Transplantation

The first study conducted by Zhong H.J. (2021) has demonstrated an antihypertensive effect of washed microbiota transplantation (WMT) in patients with HTN. According to the Consensus of Fecal Microbiota Transplantation–standardization Study Group, in WMT, microbes are extracted from the fecal matter of a healthy donor and purified via multiple cycles of automated washing, centrifuging, and filtration [50]. WMT demonstrated a short-term (3–7 days) BP-lowering effect in hypertensive subjects which was more prominent among patients who underwent WMT via the lower gastrointestinal tract and those who did not receive blood pressure medications before. The duration of even a short-term BP-lowering effect of WMT was longer in comparison with conventional antihypertensive medications [51]. 

Table 1 provides the main results obtained from studies on targeting GMB for the prevention and treatment of HTN.

## 3. Atrial Fibrillation

### 3.1. The Role of «Pro-Arrhythmic» Gut Microbiota and Its Metabolites in Atrial Fibrillation Development

#### 3.1.1. Gut Microbiota Dysbiosis Contributes to Atrial Fibrillation Susceptibility through Inflammation

In a key study that set out to determine the possible role of GMB in atrial fibrillation (AF) development, Zhang Y. et al. (2021) found that GMB dysbiosis is closely linked to atrial AF pathogenesis [52]. They were the first to demonstrate that age-related GMB alterations induce an increase in the concentration of LPS and impaired glucose tolerance, which in turn enhances atrial fibrosis and promotes AF development. It was shown that FMT from aged to young rats led to a higher AF susceptibility in the latter, and vice versa.

**Figure 2:**
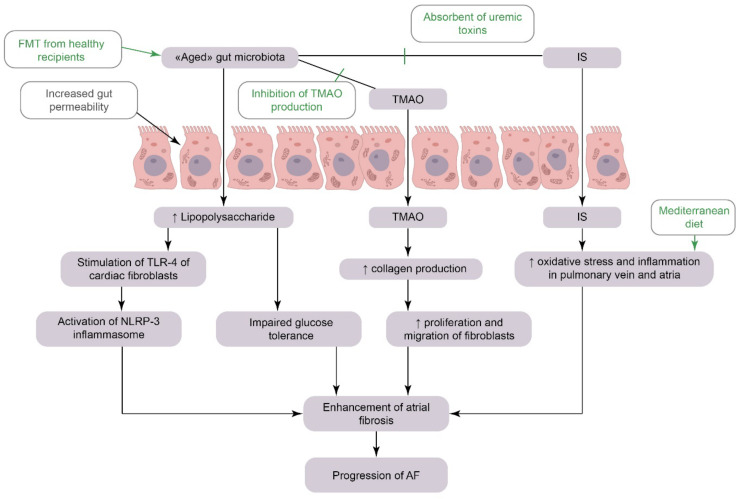
The gut microbiota-related pathways as possible targets for clinical interventions in atrial fibrillation management. AF: Atrial Fibrillation, TMAO: Trimethylamine N-oxide, IS: Indoxyl Sulfate, TLR-4: Toll-like receptor-4, FMT: Fecal Microbiota Transplantation, NLRP-3: *Pyrin* Domain Containing Protein *3*.

The mechanism for LPS-driven atrial pro-arrhythmic action could be attributed to the activation of atrial nucleotide binding and oligomerization domain-like receptor family pyrin domain-containing protein 3(NLRP3)-inflammasome. This finding is consistent with that FMT from young hosts could partially decrease both AF susceptibility and atrial fibrosis via the inhibition of atrial NLRP3-inflammasome activity [52]. Another important finding was that the expression and upregulation of NLRP3-inflammasome correlated with the progression of AF to more persistent forms [53]. Thus, inhibition of atrial NLRP3-inflammasome activity through GMB modulation seems to be a potential novel therapeutic intervention for senile arrhythmia diseases (see Figure 2).

#### 3.1.2. The Mechanistic Links between Gut Microbiota-Derived Metabolites and AF

Trimethylamine N-oxide is the well-researched microbial metabolite implicated in AF pathophysiology. In animal models of AF, TMAO has been shown to exacerbate autonomic activity and stimulate the release of inflammatory cytokines [54]. In vitro, TMAO has driven increased collagen production by the fibroblasts, thereby contributing to cardiac fibrosis development [55]. In addition, TMAO could also indirectly increase AF susceptibility through pro-atherosclerotic effects, cardiac remodeling and aortic stiffening [56]. Numerous small observational clinical studies have demonstrated that elevated serum TMAO levels were predictive of thromboembolic complications of AF [57,58].

Indoxyl sulfate (IS) is the main GMB-derived uremic toxin which is strongly linked to AF development [59]. Laboratory studies have confirmed that IS promotes pro-fibrotic, pro-hypertrophic, and pro-inflammatory cellular mechanisms in cardiomyocytes. Moreover, IS induced oxidative stress and inflammation in the pulmonary vein and atrium thereby implicating the formation of the arrhythmogenic substrates [60]. Considering that atrial interstitial fibrosis creates a substrate for AF, Aoki et al. (2015) examined whether the absorbent of uremic toxins AST-120 may have a beneficial impact on renal-dysfunction induced AF. In a rat model, treatment with AST-120 significantly decreased serum IS levels, alleviated oxidative stress, inflammation, and atrial fibrosis and, consequently, attenuated AF inducibility [61].

Lipopolysaccharide may also contribute to AF development through the acceleration of pro-fibrotic, pro-atherogenic and pro-inflammatory mechanisms. In fact, low circulating LPS levels provoke cardiac fibrosis, even without any prior myocardial injury [62]. Since toll-like receptor-4 (TLR-4) is expressed in cardiac fibroblasts, for which LPS is a ligand, these cells directly respond to LPS by activating the NLRP3 inflammasome, further contributing to inflammation and fibrosis [3]. A recent prospective observational study has shown a strong association between circulating LPS and major adverse cardiovascular events (MACE) in a large cohort of AF patients [63]. 

### 3.2. Possible Clinical Interventions to Modify Gut Microbiota Composition and Metabolites in AF Management

#### 3.2.1. Dietary Interventions

The mediterranean diet may also serve as a useful tool in the management of AF. The prospective cohort study involving 690 patients with AF showed that adherence to MedD was associated with higher antioxidant activity of glutathione peroxidase-3 (GPx3), and that low GPx3 levels predict MACE in patients with AF [64]. The major finding of another case-control study was that patients hospitalized with the first detected episode of AF in the highest quartile of Mediterranean Score, were two-fold more likely to develop spontaneous conversion to sinus rhythm compared to those in the lowest quartile [65]. Thus, MedD could represent an effective dietary approach to prevent MACE in AF patients via lowering oxidative stress.

#### 3.2.2. Targeting the Metabolic Pathway of TMAO

Targeting TMAO could represent a novel therapeutic strategy to tackle cardiac hypertrophy and fibrosis thereby exerting an anti-arrhythmic effect. Recent studies have shown that 3,3-dimethyl-1-butanol (DMB), which was detected in some balsamic vinegar, red wines, some cold-pressed extra virgin olive oils, and grape seed oils decreased serum TMAO levels. In a mice model, inhibition of TMAO production by DMB resulted in the attenuation of both structural and electrical remodeling in overload-induced heart failure [66]. Preclinical study results also demonstrated that treatment with DMB prevented Western diet-induced interstitial fibrosis and inflammation in the heart [67]. It may be concluded that reducing TMAO levels could decrease the risk of AF development via attenuation of cardiac remodeling and associated hemodynamic changes that affect the atria.

Table 2 illustrates the main findings of the forementioned studies on GMB modulation in AF.

## 4. Congestive Heart Failure

### 4.1. Gut Microbiota as a Cornerstone in the Development of Congestive Heart Failure

#### 4.1.1. “Gut Hypothesis” of Congestive Heart Failure

**Figure 3:**
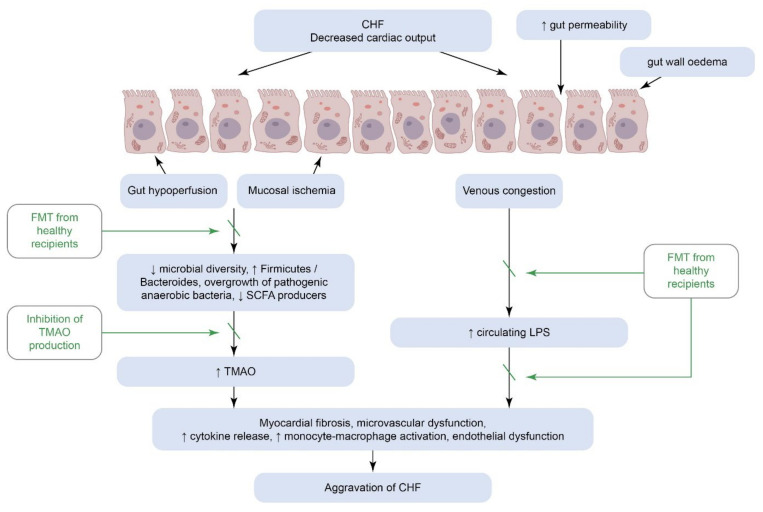
The «gut-heart axis» in the pathogenesis of congestive heart failure. CHF: Congestive Heart Failure, LPS: Lipopolysaccharide, FMT: Fecal Microbiota Transplantation, SCFA: Short Chain Fatty Acids, TMAO:Trimethylamine N-oxide.

Over the last two decades, GMB has become more and more recognizable as an important contributor to congestive heart failure (CHF), particularly immune-mediated subtypes of cardiomyopathy. Decreased cardiac output is associated with splanchnic congestion, leading to gut hypoperfusion, increased permeability of the intestinal barrier, and augmented bacterial biofilm. In CHF, gut microbiota composition is characterized by expansion of potentially pathogenic microbes and depletion of those ones with anti-inflammatory properties. The latter leads to increased bacterial metabolites (such as LPS, TMAO) translocation and their interaction with the intestinal and systemic innate immune systems thereby contributing to low-grade systemic inflammation and aggravation of CHF (see Figure 3) [68]. 

#### 4.1.2. Gut Microbiota Implication in the Initial Development of Immune-Mediated Cardiomyopathies

In a recent preclinical study, Gil-Cruz C. et al. (2019) described the critical role of gut commensals *Bacteroides thetaiotaomicron* and *B. faecis* in the autoimmune response against the cardiac cellular proteins [69]. These microbes produce an enzyme beta-galactosidase that seems to mimic cardiac myosin heavy chain 6 (MYH6). The beta-galactosidase induces the proliferation of MYH6-specific CD4+ T-cells which then infiltrate the myocardium thereby promoting inflammatory cardiomyopathy. This also accords with the finding indicating that patients with biopsy-proven myocarditis had a greater relative abundance of *B. theta* compared to healthy controls. Moreover, it has demonstrated a positive correlation between levels of *B. theta*-specific antibodies and disease progression and severity [70]. 

In a study of mice with experimentally induced autoimmune myocarditis, Hu X.-F. et al. (2019) found that FMT from untreated male mouse donors resulted in a decrease in the severity of myocarditis via the reduction in the myocardial infiltration and normalization of the GMB composition [69].

There is an unambiguous relationship between anthracycline-induced cardiotoxicity and gut epithelial damage and further GMB involvement. Antracyclines use, in particular Doxorubicin (DOX), leads to increased gut permeability for LPS. As a consequence, LPS together with TLR-4 can give rise to DOX-induced damage to the heart, kidneys, liver, and gut [71]. In order to better understand the role of GMB in DOX-induced cardiomyopathy, An L. et al. (2021) explored the effects of FMT on DOX-treated mice. According to the results, FMT has been found to significantly improve cardiac function and attenuated perivascular and interstitial fibrosis compared to DOX-treated mice, which did not undergo this procedure. FMT-treated mice exhibited a decrease in gut damage and improvement in microbial composition, function and diversity [72]. Despite these promising results from animal studies, human data are still scarce and require further investigation.

The 5-year mortality rate of 55% for HF patients indicates that there is a great need for novel prevention and treatment strategies to improve outcomes [73]. Restoring healthy GMB has become an area of therapeutic concern for patients with CHF, which consists of dietary modifications, dietary fiber intake, probiotic therapy, FMT, and TMA-lyase inhibitors.

### 4.2. Targeting the Gut Microbiome to Prevent and Treat Heart Failure

#### 4.2.1. Dietary Interventions

A healthy diet is a fundamental part of the prevention and treatment of HF due to its positive effect on the GMB. In a hypertensive mice model, modulation of GMB through a high-fiber diet along with the addition of acetate led to a significant reduction in myocardial fibrosis and hypertrophy thereby preventing the development of HF [37]. The MedD is likely to maintain optimal GMB status, improve ventricular function in HF subjects [74] and substantially decrease the incidence of HF [75]. According to PREDIMED study results, following the MedD was responsible for approximately a 30% reduction in MACE during a nearly 5-year period [76]. A systematic review and meta-analysis of RCTs with a total of 10,950 participants showed that the MedD reduced the incidence of HF by 70% [77]. Levitan et al. (2013) showed that greater adherence to the DASH-diet modestly lowered mortality in women with HF [78]. These data support the concept that adapting existing CHF guidelines is necessary with respect to dietary interventions besides sodium and fluid restriction.

#### 4.2.2. Probiotics

In a study of rats after coronary artery ligation, Gan X.T. et al. (2014) reported that probiotic administration has a positive effect on the outcomes of myocardial infarction-induced HF and provides a direct benefit to cardiac tissue. Administration of *L. rhamnosus GR-1* and *L. plantarum 299v* led to significant improvement in hemodynamic parameters and structural abnormalities. Probiotic administration near-normalized several parameters of HF including cardiac output, stroke volume, left ventricle weight, ejection fraction, fractional shortening, etc. One unanticipated finding was that probiotic supplementation did not induce significant changes to the microbial composition of the gut [79]. Small RCT evaluated the effect of probiotic *S. boulardii* in 20 patients with systolic CHF. Obtained results demonstrated a reduction in biochemical and serum inflammatory biomarkers such as creatinine, uric acid, high-sensitive C-reactive protein, and also an improvement in echocardiographic parameters (left atrial diameter, left ventricle ejection fraction) [80]. 

#### 4.2.3. Lowering TMAO Could Break the Vicious Circle

The regulation of the harmful metabolites’ production by the GMB in CHF is an extensive area of research. In mice with overload-induced HF, treatment with DMB resulted in the inhibition of TMA formation with subsequent reduction in TMAO-level. The second major finding was that DMB-treated mice showed attenuation of left ventricle structural remodeling and reduced expression of pro-inflammatory cytokines in the heart [67].

In a murine model of HF, both dietary TMAO removal and administration of choline TMA lyase inhibitor, iodomethylcholine, significantly lowered serum TMAO levels and subsequently led to the mitigation of unfavorable cardiac remodeling and fibrosis [81]. The results of this research support the idea that blocking TMAO production via the choline TMA-lyase inhibitors may serve as a reasonable therapeutic approach to managing HF. Another promising strategy is to conduct tailored monitoring of TMAO and to provide dietary advice based on health conditions, as well as individual characteristics such as food and cultural preferences.

### 4.3. The GutHeart Trial Results

The Targeting Gut Microbiota to Treat Heart Failure (GutHeart) was the first comprehensive trial focused on GMB as a potential therapeutic target in HF. The purpose of the GutHeart trial was to determine whether targeting GMB with either probiotics Saccharomyces boulardii or the non-absorbable antibiotic Rifaximin could affect cardiac function in well-treated HF with reduced ejection fraction [82]. All patients were in a well-compensated state and had mild symptoms. Contrary to expectations, this study did not find a significant difference in LVEF, 6-min walk distance, GMB diversity, serum TMAO and inflammatory markers levels, after 3 months of intervention in both rifaximin and *Saccharomyces boulardii* groups. A possible explanation for the lack of clinical effect may be a low degree of dysbiosis at baseline, measured by the microbial diversity in well-treated patients with HF. In addition, short-term studies such as GutHeart do not necessarily show subtle changes over time.

Previously published papers described differences in the GMB composition and GMB-derived metabolites among patients during both decompensation and compensation phases of CHF. Notably, patients exhibited intestinal overgrowth of pathogenic bacteria including *Shigella*, *Campylobacter*, and *Salmonella* in relation to HF severity [83]. Hayashi T. et al. (2018) also reported an increased abundance of the genera *Escherichia*/*Shigella* in the setting of decompensated HF [84].

The most obvious finding to emerge from the GutHeart trial is that broad interventions with probiotics and antibiotics might be insufficient to markedly affect the GMB in the setting of well-compensated HF. Researchers also suggested that properly treated HF is not always accompanied by GMB dysbiosis. Overall, these results indicate that a more precise approach focusing on either specific bacterial taxa or gut-derived metabolites might be considered. Although extensive research has been carried out in patients with clinically stable heart failure, no single study exists that examined the effect of GMB modulation in a decompensated state. 

Data from studies regarding GMB modulation in heart failure are shown in Table 3.

## 5. Conclusions

This paper provides an overview of the results of the latest studies on GMB modulation for the prevention and treatment of arterial hypertension, atrial fibrillation and congestive heart failure. Considering the great strides made in understanding the role of GMB in the pathophysiology of these CVDs, there is a tremendous potential for developing novel GMB-based therapies which include personalized dietary interventions, supplementation with prebiotics and probiotics, using specific postbiotics, TMAO-inhibitors and FMT. Several questions still remain to be answered. Whether it is necessary or not to assess GMB status before prescribing GMB-based therapies? Should GMB modulation be considered at early or advanced disease stage or both? More research using controlled trials is needed to determine clearly defined indications for each option of GMB modulation. Further studies need to be carried out in order to evaluate the efficacy and safety of all GMB-based therapeutic approaches in these CVDs. The extensive pre-clinical and clinical studies have promoted our understanding of the clinical application of gut microbiota-targeting therapy and opened up new therapeutic opportunities for CVDs. To sum up, the issue of targeting GMB is an intriguing one that could be thoroughly explored in further research.

## Figures and Tables

**Table 1:** The main results of research on targeting gut microbiota and its metabolites in arterial hypertension.

Patients/Model	Intervention	Main Findings	Ref
Rat model	Every other day fasting (EODF); cholic acid supplementation	EODF ↓ systolic BP, significantly altered the community structure of GMB and ↑ BA metabolism genes	[29]
Murine model	High-fiber diet, acetate supplementation	↓ *Firmicutes*/*Bacteroidetes* ratio, ↑ the abundance of *Bacteroides acidifaciens*; ↓ systolic and diastolic BPs, cardiac fibrosis, and left ventricular hypertrophy; acetate also markedly ↓ renal fibrosis.	[37]
Murine model	Propionate intake in drinking water	Attenuation of cardiac hypertrophy, fibrosis, vascular dysfunction, ↓ susceptibility to ventricular arrhythmias; ↓ aortic atherosclerotic lesion area.	[46]
Murine model	Butyrate administration	↓ mean arterial pressure; ↓ spontaneous cardiac baroreceptor reflex gain and ↑ cardiac sympathetic tone in Ang II-treated mice; Improvement of gut barrier function, acceleration of mucus turnover and amelioration of gut inflammation.	[47]
Murine model	Diet lacking prebiotic fiber with or without addition SCFAs	In the absence of dietary fiber, SCFAs supplementation protected against the development of HTN, myocardial hypertrophy and fibrosis.	[49]
145 untreated hypertensive patients	Sodium restriction to 2000 mg/day	↑ of circulating SCFAs; ↓ BP and improved arterial compliance	[27]
1422 subjects	Intermittent fasting	The mean values for the whole cohort ↓ dramatically (from 131.6 ± 0.7 to 120.7 ± 0.4 for systolic BP and from 83.7 ± 0.4 to 77.9 ± 0.3 for diastolic BP (fasting intervention: *p* < 0.001 for both); the effect size was directly related to fasting duration (*p* < 0.001) without gender difference	[30]
Patients with metabolic syndrome	5-day fasting + DASH-diet/DASH diet alone	Fasting + DASH diet substantially ↓ 24 h ambulatory systolic BP and mean arterial pressure 3 months post-intervention as opposed to DASH diet alone. Participants who were prescribed fasting + DASH diet have been shown to ↓ their intake of antihypertensive medication by 3 months post-intervention in comparison to counterparts who followed a DASH diet only.	[31]
5 studies involving 233 participants	Inulin-type carbohydrate supplementation	↓ systolic BP in women (WMD: −12.19 mmHg; 95% CI: −17.25, −7.13, *p* = 0.012).	[39]
23 RCTs involving 2037 participants	Probiotic consumption	Probiotics ↓ systolic BP only in HTN [WMD = −3.31 mmHg, 95% CI: −5.71, −0.92; *p* = 0.007] and type 2 diabetes mellitis (WMD = −4.85 mmHg, 95% CI: −9.28, −0.42; *p* = 0.032); Probiotics ↓ diastolic BP only in HTN (WMD = −2.02 mmHg, 95% CI: −3.68, −0.36; *p* = 0.017). BP-lowering effect could be observed for a short-term time 8–10 weeks;	[40]
352 community-living initially normotensive subjects	Fermented milk products containing *Lactobacillus casei strain Shirota*	BP was more likely to remain in normal range over 5 years in participants who took ≥3 fermented milk products rather than <3 times/week (relative risk 0.398 [95% confidence interval 0.167–0.948], *p* = 0.037).	[42]
260 patients (187 normotensive patients and 73 patients with HTN)	WMT	↓ systolic BP: −5.09 ± 15.51, (*p* = 0.009); ↓ diastolic BP: −7.74 ± 10.42, (*p* < 0.001). WMT via the lower gastrointestinal tract and those not taking antihypertensive drugs had a greater decrease in systolic BP, and hypertensive patients not taking antihypertensive drugs also had a greater decrease in diastolic BP. WMT increased the Shannon Diversity Index in 6 of 8 hypertensive patients.	[51]

**Table 2:** The main results of studies on manipulation of gut microbiota in atrial fibrillation.

Patients/Model	Intervention	Main Findings	Ref
Rat model	FMT	FMT from aged AF-rats to young rats ↑ AF susceptibility, atrial fibrosis, ↑ inducibility and longer AF-duration; FMT from aged to young rats resulted in gut barrier dysfunction; Transferring GMB from young to aged rats ↓ the activation of atrial NLRP3-inflammasome and ↓ atrial fibrosis; also, the aged rats that were re-colonized with young microbiota failed in inducing AF by burst electrical pacing.	[52]
Rat model	AST-120	↓ renal dysfunction-induced oxidative stress, inflammation, and atrial fibrosis; ↓AF inducibility.	[61]
Pressure-overload-induced HF mice	Normal diet and given water supplemented with or without 1.0% DMB for 6 weeks	↓ TMAO levels in overload-induced HF mice, ↓ adverse cardiac structural and electrical remodeling.	[67]
690 AF-patients	Mediterranean diet	↑ antioxidant activity of GPx3 in AF, ↓ vascular events rate.	[65]

**Table 3:** The main results of research on modulation of gut microbiota and its metabolites for the prevention and treatment of congestive heart failure.

Patients/Model	Intervention	Main Findings	Ref
EAM mouse	FMT	↓ myocardial damage, ↓ inflammatory infiltration with cardiomyocyte disarray and necrosis in the cardiac tissues. ↑ increasing the Bacteroides population, ↓ *Firmicutes*/*Bacteroidetes* ratio and reshaping the GMB composition;	[71]
DOX-treated mice	FMT	Improvement of cardiac function, ↓ of DOX- induced GMB dysbiosis,↑ colorectum length, ↓ goblet cell loss, number of intestinal ulcers and lymphocyte cluster infiltration, loss of tight junction protein, and ↓ plasma LPS level.	[73]
Murine model	Normal diet or a Western diet (WD), without or with 1.0% DMB in drinking water	↓ cardiac dysfunction and fibrosis in mice fed a WD by inhibition WD-induced increase in serum TMAO level.	[68]
Rat model	Administration of the probiotic *Lactobacillus rhamnosus* GR-1 or placebo in the drinking water	Significant attenuation of left ventricular hypertrophy, improvement of hemodynamic parameters such as preservation of left ventricular ejection fraction and fractional shortening.	[80]
218 HF-patients	Mediterranean diet	Improvement of biventricular systolic function.	[75]
32,921 women	Mediterranean diet	A high adherence to the MedD ↓ risk of myocardial infarction (RR: 0.74, 95% CI: 0.61–0.90, *p* = 0.003), HF (RR: 0.79, 95% CI: 0.68–0.93, *p* = 0.004) and ischemic stroke (RR: 0.78, 95% CI: 0.65–0.93, *p* = 0.007)	[76]
3215 women experienced a HF hospitalization	Mediterranean and Dietary Approaches to StopHypertension (DASH) diet	Higher adherence to the Mediterranean diet was associated with a ↓ hazard rate of death among women with HF; Women in the top quartile of the DASH diet score had a 16% ↓ hazard rate of death than those in the bottom quartile (*p* for linear trend = 0.01).	[79]
150 patients with stable HFrEF	Rifaximin or *Saccharomyces boulardii*	No significant effect on LVEF, microbiota diversity, plasma C-reactive protein and TMAO levels.	[84]

## Data Availability

Not applicable.
